# Pathogenesis of multiple sclerosis: expression of HERV-Fc1: a human endogenous retrovirus

**DOI:** 10.1186/1742-4690-8-S2-O23

**Published:** 2011-10-03

**Authors:** Magdalena Janina Laska, Tomasz Brudek, Kari Konstantin Nissen, Tove Christensen, Anne Møller Larsen, Thor Petersen, Bjørn Andersen Nexø

**Affiliations:** 1 Department of Biomedicine, Aarhus University, 8000 Aarhus C, Denmark; 2 Research Laboratory for Stereology and Neuroscience, Bispebjerg University Hospital, 2400 Copenhagen NV, Denmark; 3 Department of Neurology, Aarhus University Hospital, 8000 Aarhus C, Denmark

## Background

Multiple Sclerosis (MS) is considered to be an autoimmune disease with unknown cause and with immune system dysregulation. Among environmental factors, viruses are most often connected with the etiology of MS.

Human Endogenous Retroviruses (HERVs) constitute 5-8% of human genomic DNA and have been detected as transcripts and proteins in the central nervous system (CNS) and peripheral blood, frequently in the context of neuro inflammation [1,2].

In our recent genetic epidemiology study in MS patients [3] we found indications of a specific genetic association between HERV-Fc1 (which belongs to the HERV-H/F family) and MS. One marker neighboring HERV-Fc1 (rs391745) had a *p*-value of 1.3x10^-6^ for disease association in a trend test.

## Materials and methods

Using polyclonal rabbit antibodies raised against a Gag peptide, conserved in the HERV-H/F family, the authors studied the expression of a capsid protein of HERV-H/F origin by flow cytometry in PBMCs from healthy controls and from MS patients with non-active or active disease. Furthermore, we have undertaken the first rigorous SYBR green-based absolute Q-PCR evaluation approach to quantify extracellular HERV-Fc1 RNA viral loads in plasma from patients with MS and healthy controls.

## Results

We showed significant differences in the expression of HERV-H/F Gag protein epitopes between PBMCs from healthy controls and from MS patients. We found higher HERV-H/F Gag expression in peripheral blood CD19+ cells and significantly higher expression in monocytes in patients with non-active MS than in healthy controls. For patients with active MS, much higher differences in the HERV-H/F Gag expression relative to healthy controls were detected for CD4+ and CD8+ T cells indicating that this HERV family may play a role in the extensive activation of the innate and adaptive immune responses in MS. Also for patients with active MS, the expression of HERV-H/F Gag by CD4+ T cells was significantly higher than that by CD8a+ T cells.

Extracellular HERV-Fc1 RNAs were detected in plasma samples from both healthy controls and from MS patients. In plasma samples from patients with non-active MS, higher levels of extracellular HERV-Fc1 RNAs were found as compared with healthy controls. Moreover, a 5-fold increase of the amount of extracellular HERV-Fc1 RNA was found in plasma samples from patients with active MS as compared with healthy controls.

## Conclusions

These findings strengthen the link between HERV-Fc1 and the pathology of MS. The cause and biological consequences of these differential expressions will be the subject of further investigation. HERV-Fc1 biology could be a compelling area for understanding the pathology of MS and possibly other autoimmune disorders.
